# Preprotection of Tea Polysaccharides with Different Molecular Weights Can Reduce the Adhesion between Renal Epithelial Cells and Nano-Calcium Oxalate Crystals

**DOI:** 10.1155/2020/1817635

**Published:** 2020-01-06

**Authors:** Yao-Wang Zhao, Li Liu, Chuang-Ye Li, Hui Zhang, Xin-Yuan Sun, Jian-Ming Ouyang

**Affiliations:** ^1^Department of Urology, Hunan Children's Hospital, Changsha 410007, China; ^2^Institute of Biomineralization and Lithiasis Research, Jinan University, Guangzhou 510632, China

## Abstract

Crystal adhesion is an important link in the formation of kidney stones. This study investigated and compared the adhesion differences between nano-calcium oxalate monohydrate (COM) and human renal proximal tubule epithelial (HK-2) cells before and after treatment with tea polysaccharides (TPSs) TPS0, TPS1, TPS2, and TPS3 with molecular weights of 10.88, 8.16, 4.82, and 2.31 kDa, respectively. TPS treatment effectively reduced the damage of COM to HK-2 cells, thereby resulting in increased cell activity, decreased release of lactate dehydrogenase, cell morphology recovery, decreased level of reactive oxygen species, increased mitochondrial membrane potential, increased lysosomal integrity, decreased expression of adhesion molecule osteopontin and eversion of phosphatidylserine, and decreased crystal adhesion. Among the TPSs, TPS2 with moderate molecular weight had the best protective effect on cells and the strongest effect on the inhibition of crystal adhesion. Thus, TPS2 may be a potential anticalculus drug.

## 1. Introduction

Tea is one of the most popular drinks in the world [1] and has many variations, such as green, black, oolong, and Pu'er tea. Numerous studies have shown that tea has many properties, as follows: antioxidant property, cholesterol-lowering property, inhibition of hypertension, inhibition of blood coagulation, dissolution of fibrinogen, reduction of endothelin levels, activation of GSH-Px, protection of LDL oxidation, prevention of cardiovascular disease, and anticancer property [2–6]. Different concentrations (0.00078–5 *μ*g/mL) of black tea extract (BTE) have toxic effects on human colon cancer cells (HT-29), human breast cancer cells (MCF-7), and human alveolar cancer cells (A549) and have no effect on normal cells (NIH-3T3) [4]. BTE can induce DNA strand breakage and oxidative damage in HT-29 and MCF-7 cancer cells. San Cheang et al. [5] prevented the increase of ER stress markers and reactive oxygen species (ROS) levels and the downregulation of Hcy metabolic enzymes in the aortae of Ang II-infused rats through BT treatment. Fei et al. [6] observed the that water extract of Pu'er tea, BT, and green tea increased the lifespan of worms, postponed A*β*-induced progressive paralysis of Alzheimer's disease in transgenic worms, and improved the tolerance of worms to oxidative stress induced by heavy metal Cr^6+^.

The main component of kidney stones is calcium oxalate (CaOx) [7]. The antistone effect of tea has become the focus of researchers [8–11]. Alhaji herbal tea prevents the formation of CaOx kidney stones at high concentrations of calcium and oxalate ions [8]. *Blumea balsamifera* (sambong) tea can form small stones that can be easily eliminated through urination because of the decrease in surface free energy and increase in nucleation rate [9]. Rode et al. [10] showed that the prevalence of CaOx monohydrate (COM) stones immensely decreases among green tea drinkers in a population of 273 hypercalciuric stone formers. Chen et al. [11] evaluated 13842 subjects with kidney stones through ultrasound and observed that the amounts of daily tea consumption are 119.2 ± 306.8 and 131.7 ± 347.3 mL in groups with and without renal stone disease, respectively. Daily tea consumption ≥ 240 mL (two cups) is associated with a low risk of renal stone disease. These beneficial effects of tea are attributed to its active ingredients, as follows: polysaccharides (PSs), polyphenols, alkaloids, amino acids, vitamins, and inorganic elements [12]. However, the antistone mechanism of tea PSs (TPSs) has not been fully elucidated.

In our previous study [13], we investigated the antioxidant activities of four green TPSs with different molecular weights (10.88 (TPS0), 8.16 (TPS1), 4.82 (TPS2), and 2.31 kDa (TPS3)) and their repair of damaged human renal proximal tubule epithelial (HK-2) cells. Four TPSs repaired mitochondria, lysosomes, and intracellular DNA in HK-2 cells, and TPS2 had the strongest ability.

The prevention of kidney stones is more important than clinical treatment [14–16]. In our previous studies, we have found that polysaccharides extracted from green tea [13, 17] and *Porphyra yezoensis* [18] have the ability of repairing damaged renal epithelial cells. The cells repaired by polysaccharide inhibited the adhesion of CaOx crystals and promoted the endocytosis of the adherent crystals. Cell repair is to repair damaged renal epithelial cells so as to prevent the formation of kidney stones, which is a passive treatment method. However, for undamaged cells, protecting cells from oxidative damage of urine crystallites or oxalic acid *in vivo* in advance is an active effective method to prevent kidney stone formation, and its clinical value is greater than that of passive repair. TPSs with good antioxidant capacity may protect cells and increase their ability to resist oxidative damage. On this basis, this study investigated the adhesion of CaOx crystals to renal epithelial cells before and after protection by TPSs with different molecular weights, in order to provide insights into the active prevention of the formation of kidney stones and investigation of new antistone drugs.

## 2. Experimental Methods

### 2.1. Reagents and Instruments

Tea polysaccharide (TPS0) was provided by Shaanxi Ciyuan Biological Co., Ltd. and its molecular weight is 10.88 kDa. The degradation of polysaccharides was performed as previously described [13, 17]. The molecular weights of TPS1, TPS2, and TPS3 were 8.16, 4.82 and 2.31 kDa, respectively.

Calcium oxalate monohydrate (COM) was synthesized according to the previous reference [19]. SEM and XRD indicate that it is a target crystal with a size of about 100 nm.

Human kidney proximal tubular epithelial (HK-2) cells were purchased from the Shanghai Cell Bank, Chinese Academy of Sciences (Shanghai, China). Fetal bovine serum and cell culture medium (DMEM-F12) were purchased from HyClone Biochemical Products Co. Ltd. (Beijing, China). A cell proliferation assay kit (Cell Counting Kit-8, CCK-8) was purchased from Dojindo Laboratory (Kumamoto, Japan). Acridine orange (AO), hematoxylin and eosin staining kit, reactive oxygen detection kit (DCFH-DA), lactate dehydrogenase (LDH) kit, 5,5′,6,6′-tetrachloro-1,1′,3,3′-tetraethylbenzimi-dazolylcarbocyanine iodide (JC-1), osteopontin primary antibody (OPN), rabbit anti-rat (FITC-IgG), Annexin V-FITC/PI apoptosis detection kit, cell membrane red fluorescent probe (DiI), and 4′,6-diamidino-2-phenylindole (DAPI) were all purchased from Shanghai Beyotime Bio-Tech Co., Ltd. (Shanghai, China). The paraformaldehyde and ethanol are of analytical grade (Guangzhou Chemical Reagent Factory).

The apparatus included a laser confocal microscope (LSM510 META DuoScan, ZEISS, Germany), optical microscope (OLYMPUS, CKX41, Japan), microplate reader (SafireZ, Tecan, Switzerland), multifunction microplate detector (Synergy H1M, BioTek, USA), and flow cytometer (BD FACSAria, USA).

### 2.2. Cell Culture and Experimental Model

According to our previous study [17], HK-2 cells were cultured in a DMEM-F12 culture medium containing 10% fetal bovine serum and 100 U/mL penicillin-100 *μ*g/mL streptomycin antibiotics with pH 7.4 at 37°C in a 5% CO_2_ humidified environment. Upon reaching an 80%–90% confluent monolayer, cells were blown gently after trypsin digestion to form cell suspension for the following cell experiment.

Cell suspension with a cell concentration of 1 × 10^5^ cells/mL was inoculated with 200 *μ*L, 1 mL, and 2 mL/well in 96-, 12-, and 6-well plates, respectively, and incubated in DMEM-F12 culture medium for 24 h. The cells were divided into three groups:

1. Normal control group, in which only a serum-free culture medium was added
2. Damage control group, in which a serum-free culture medium with 200 *μ*g/mL COM was added and incubated for 6 h
3. Protection group, in which the crystal was pretreated with 80 *μ*g/mL tea polysaccharide for 1 h, then the serum-free medium of 200 *μ*g/mL COM was added to the cell for 6 h

### 2.3. Cell Viability Detection by CCK-8

The experimental model is the same as in Section 2.2. After reaching the time, add 10 *μ*L of CCK-8 reagent to each well of the 96-well plate and incubate for 4 h. The OD values were measured using a microplate reader instrument at 450 nm to detect the repair capacity of polysaccharides.

### 2.4. Lactate Dehydrogenase (LDH) Release Assay

Divide the cells into the following groups:

1. Cell-free medium (background blank wells)
2. Untreated cell wells for subsequent lysis (sample maximum enzyme activity control wells)
3. Normal control group, in which only a serum-free culture medium was added
4. Damage control group, in which a serum-free culture medium with 200 *μ*g/mL COM was added and incubated for 6 h
5. Protection group, in which the crystal was pretreated with 80 *μ*g/mL tea polysaccharide for 1 h, then the serum-free medium of 200 *μ*g/mL COM was added to the cell for 6 h

After the damage was completed, each group of 96-well plates was assayed for OD using a microplate reader according to the LDH kit test method to determine the repair ability of the polysaccharide. The specific operation is as follows: add 60 *μ*L LDH detection working solution to each well, mix well, and incubate in the dark (about 25°C) for 30 minutes (can be wrapped in aluminum foil and placed in a horizontal shaker or shaken on a rocking bed). Absorbance was then measured at 490 nm. Dual-wavelength measurements are performed using any wavelength of 600 nm or greater than 600 nm as the reference wavelength. The measured absorbance of each group should subtract the absorbance of the background blank control wells. The result was calculated as follows: LDH release (%) = (absorbance of the treated sample − absorbance of the sample control well)/(absorbance of the cell's maximum enzyme activity − absorbance of the sample control well) × 100.

### 2.5. Hematoxylin and Eosin (HE) Staining

According to our previous study [13], cell morphology was observed by HE staining. The experimental model is the same as in Section 2.2. After the treatment time, the cells of the 12-well plate were fixed with 4% paraformaldehyde for 15 min at room temperature. Then, the cells were stained with hematoxylin stain and incubated for 15 min. Then, cells were washed with distilled water for 2 minutes to remove excess stain. After that, the cells were stained with eosin staining solution for 5 min. The cells were washed with distilled water for 2 minutes to remove excess eosin. After treatment, the cells in the 12-well plate were observed under the optical microscope: the cell nucleus was stained purple or blue and the cytoplasm was stained pink or red.

### 2.6. Reactive Oxygen Species (ROS) Detection

According to our previous study [13], the ROS level was analyzed by DCFH-DA staining. The experimental model is the same as in Section 2.2. After reaching the incubation time, the cells were then stained with 500 *μ*L DCFH-DA with a dilution ratio of 1 : 1000 in a serum-free medium and incubated for 30 min, then washed twice with PBS; the slides of cells in the 12-well plate were observed with a fluorescence microscope and the fluorescence intensity of cells in the 96-well plate was detected with a multifunction microplate detector.

### 2.7. Measurement of Mitochondrial Membrane Potential (*ΔΨ*m)

According to our previous study [13], the *ΔΨ*m was analyzed by JC-1 staining. The experimental model is the same as in Section 2.2. After reaching the incubation time, the supernatant was aspirated and the cells were washed twice with PBS. Finally, the samples were stained with JC-1 for 15 min. Then, the cells were washed twice with PBS. The cells in the 12-well plate were observed with an optical microscope, and the fluorescence intensity of cells in the 96-well plate was detected with a multifunction microplate detector.

### 2.8. Lysosomal Integrity Assay

According to our previous study [13], the lysosomal integrity was detected by fluorescence staining. After the cells were then loaded with 5 *μ*g/mL AO in DMEM for 15 min, the experimental model is the same as Section 2.2. After the damage is finished, the 12-well plate was observed with a fluorescence microscope and the 96-well plate was detected with a microplate reader with excitation at 485 nm and emission at 530 (green cytoplasmic AO) and 620 nm (red lysosomal AO).

(1) $$\begin{array}{c} \text{Normal lysosomal integrity}=\frac{\text{total red fluorescence intensity}}{\text{total green fluorescence intensity}},\\ \text{Lysosomal integrity}=\frac{\text{total red fluorescence intensity}}{\text{total greenfluorescence intensity}\times \text{normal lysosomal integrity}}. \end{array}$$

### 2.9. Osteopontin (OPN) Expression Detection

According to our previous study [17], the OPN expression was detected by fluorescence staining. The experimental model is the same as Section 2.2. After reaching the damage time, 4% paraformaldehyde was added to fix the cells for 10 min. Subsequently, the sheep serum was added for 20 min. The first antibody of OPN (1 : 100) was dropped into this sample, and it was laid still overnight at 4°C. After that, the cells were rinsed three times with PBS before the addition of FITC-IgG (1 : 100) in the dark. The cells were rinsed three times with PBS again after the incubation for 0.5 h at 37°C. Finally, the cells were stained with DAPI. The 12-well plate was observed using a laser confocal fluorescence microscope. The color of the nucleus was blue, the OPN was green.

#### 2.9.1. Quantitative Detection of OPN

Referring to the above method, a 96-well plate was used to quantitatively detect the fluorescence intensity with a multifunctional microplate reader.

### 2.10. Phosphatidylserine (PS) Eversion Detection

According to our previous study [18], the PS eversion was detected by flow cytometry. The experimental model is the same as in Section 2.2. After the damage time was completed, 100 *μ*L of binding buffer and 10 *μ*L of FITC-labeled Annexin V were added, and the cells were kept in the dark for 30 min at room temperature. After treatment, the cells of the 6-well plate were detected by flow cytometry.

### 2.11. Observation of Crystal Adhesion by SEM

According to our previous study [18], the crystal adhesion was observed by SEM. The experimental group is the same as in Section 2.2. After cells are incubated with crystals for 6 h, they are fixed at 4° with 2.5% glutaraldehyde for 24 h, washed 3 times with PBS solution, dehydrated with gradient ethanol (30%, 50%, 70%, 90%, and 100%), dried at the critical point of CO_2_, and sprayed with gold. Cell morphology and crystal adhesion were observed under SEM.

### 2.12. Quantitative Analysis of the Percentage of Cells Adhered by Crystals by Flow Cytometry

According to our previous study [18], the percentage of cells with adhered crystals was assessed by flow cytometry. The experimental model is the same as in Section 2.2. The 6-well plate was precooled for 30 min at 4°C, then 200 *μ*g/mL FITC-labeled nano-COM crystals was added. After 6 h at 4°C, the cells were cultured and washed twice with cold PBS, and the percentage of cells adhering to the crystals was detected by flow cytometry.

### 2.13. Statistical Analysis

Experimental data were expressed as mean ± SD from at least three independent experiments. The experimental results were statistically analyzed using SPSS 13.0 software, and Tukey test was used to analyze the differences between the mean of each experimental group and the control group. If *p* < 0.05, there was significant difference; if *p* < 0.01, the difference was extremely significant; and if *p* > 0.05, there was no significant difference.

## 3. Results

### 3.1. Cell Viability and Lactate Dehydrogenase (LDH) Release

**Figure 1:**
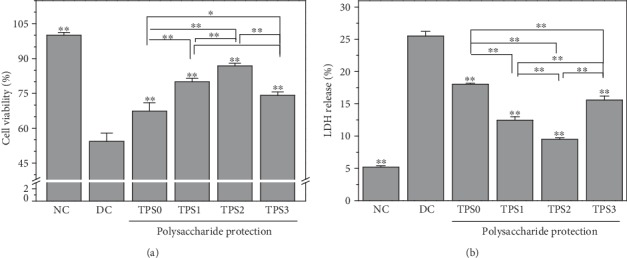
Effect of nano-COM crystals on HK-2 cell viability (a) and LDH release (b) before and after TPS protection. NC: normal control group; DC: damage control group. Polysaccharide concentration: 80 *μ*g/mL; protection time: 1 h. Nano-COM concentration: 200 *μ*g/mL; crystal damage time: 6 h. Compared with DC group, ^∗^*p* < 0.05 indicates a significant difference; ^∗∗^*p* < 0.01 indicates a very significant difference.

The toxicity of COM crystals to HK-2 cells before and after treatment of TPSs (TPS0, TPS1, TPS2, and TPS3) with molecular weights of 10.88, 8.16, 4.82, and 2.31 kDa, respectively, was compared. The changes of cell viability are shown in Figure 1(a). After 6 h incubation with nano-COM crystals, cell viability significantly decreased (54.34%). TPS treatment attenuated the toxicity of COM crystals to cells. TPS2 had the strongest protective ability, and the cell viability of TPS0, TPS1, TPS2, and TPS3 protection groups was 67.35%, 80.03%, 86.80%, and 74.13%, respectively.

LDH release is used as an important indicator of cell membrane integrity and is widely used in cytotoxicity assays [20].  Figure 1(b) shows the effect of COM crystals on LDH release from HK-2 cells before and after treatment with four TPSs. LDH release of the TPS protection group (9.48%–18.03%) was greater than that of the normal group (5.18%) but smaller than that of the damage group (25.50%), thereby indicating that the damage of HK-2 cells caused by nano-COM can be significantly inhibited by TPSs.

### 3.2. Cell Morphology

**Figure 2:**
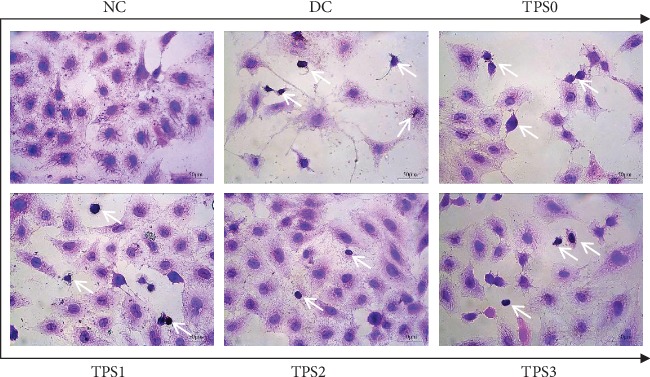
Effect of nano-COM crystals on the morphology of HK-2 cells before and after TPS protection. The white arrow is the condensed nuclei. The experimental conditions are the same as in Figure 1.

Figure 2 shows the effect of nano-COM crystals on the morphology of HK-2 cells before and after treatment of TPSs. The nucleus of normal cells is uniform, and the connection between the cells is complete. After damage of COM crystals for 6 h, the number of cells significantly decreased, the nucleus was pyknotic, the morphology was disordered, and the connection among cells was destroyed. However, the degree of cell damage was reduced after TPS treatment. The number of cells in the protection group was significantly higher than that in the damage group. The number of nucleated cells was less than in the injury group.

### 3.3. ROS Level

**Figure 3:**
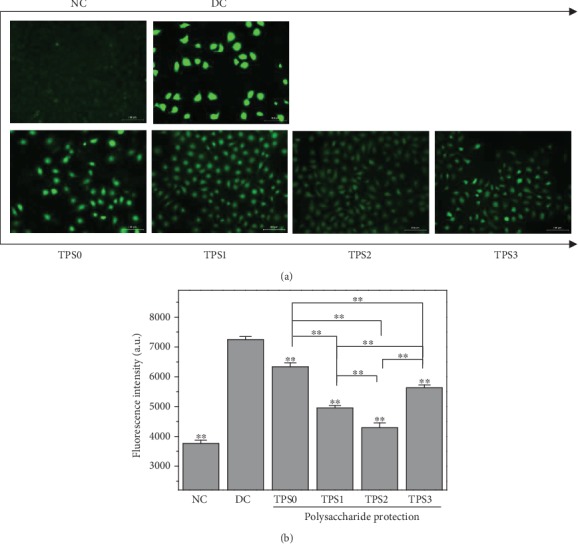
Effect of nano-COM crystals on ROS levels of HK-2 cells before and after TPS protection: (a) fluorescence observation; (b) quantitative histogram. Experimental conditions are the same as in Figure 1.

Figure 3 shows the effect of nano-COM crystals on ROS levels of HK-2 cells before and after TPS treatment. ROS fluorescence of the normal group was low. ROS fluorescence significantly increased after damage, thereby indicating the elevated ROS level. ROS fluorescence intensity of the protection group was between those of the normal and injury groups, indicating that TPSs can protect cells from damage. The fluorescence intensity quantification results (Figure 3(b)) are consistent with the results of fluorescence microscopy.

### 3.4. Mitochondrial Membrane Potential (*ΔΨ*m)

**Figure 4:**
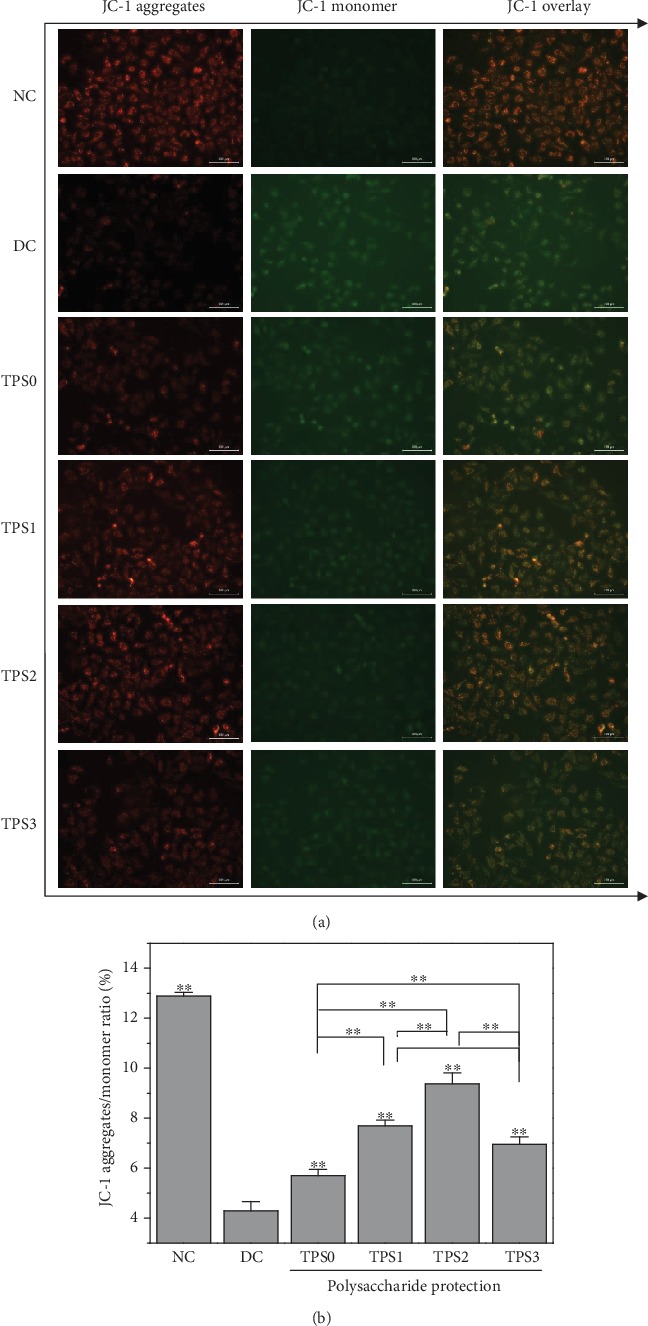
Effect of nano-COM crystals on mitochondrial membrane potential of HK-2 cells before and after TPS protection: (a) fluorescence microscopy; (b) quantitative histogram. Experimental conditions are the same as in Figure 1.

Figure 4 shows the *ΔΨ*m change of HK-2 cells before and after TPS treatment. *ΔΨ*m of normal group cells was high, and the fluorescent probe molecule JC-1 was aggregated in the matrix of the mitochondria to form a polymer (J-aggregates), thereby producing red fluorescence. *ΔΨ*m of damaged cells significantly decreased, and JC-1 mainly existed as a monomer, which produced green fluorescence. TPS treatment inhibited the decrease of *ΔΨ*m caused by COM crystals. *ΔΨ*m of protected cells was between those of the normal and injury groups. TPS2 had the strongest protective ability among the TPSs.

### 3.5. Lysosomal Integrity

Weak basic acridine orange can enter the lysosome through the cell membrane. It combines with acid hydrolase to produce orange-red fluorescence and emits a green fluorescence in the cytoplasm. The integrity of lysosomes can be detected by measuring the intensity ratio of red and green fluorescence [21]. Low amount of red fluorescence indicates more severe lysosomal damage and higher degree of cell necrosis.

**Figure 5:**
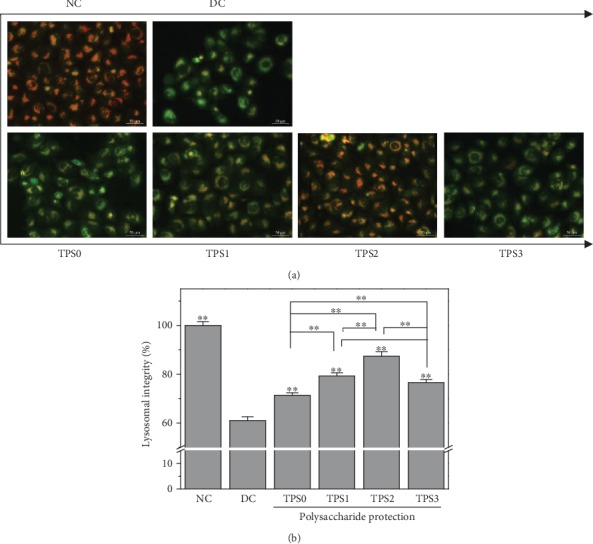
Effect of nano-COM crystals on the lysosomal integrity of HK-2 cells before and after TPS protection: (a) fluorescence observation; (b) quantitative histogram. Experimental conditions are the same as in Figure 1.

As shown in Figure 5, the lysosomal integrity of the normal group was 100.01% and that of the damage group was reduced to 60.97%. The lysosomal integrity of the cells in the protection group improved and reached 71.38%–87.32%.

### 3.6. Osteopontin (OPN) Expression

**Figure 6:**
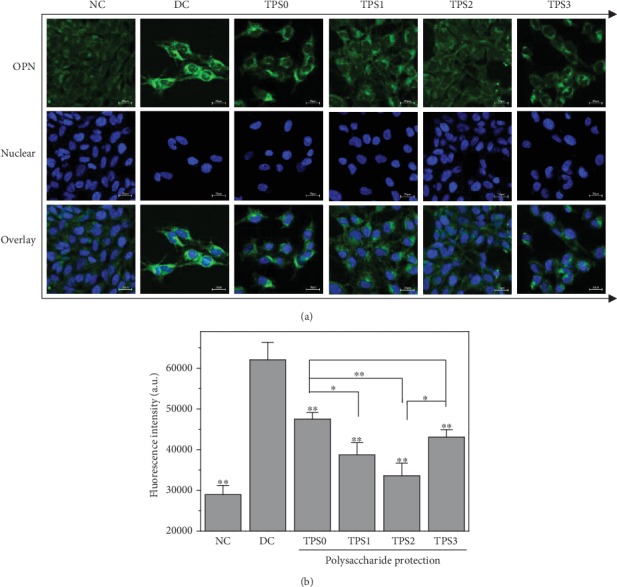
Effect of nano-COM crystals on OPN expression in HK-2 cells before and after TPS protection: (a) fluorescence observation; (b) quantitative histogram. Experimental conditions are the same as in Figure 1.

Figure 6 shows the effect of COM crystals on OPN expression of HK-2 cells before and after TPS treatment. The green fluorescence of the normal group was not obvious, whereas in the injury group was significantly enhanced, thereby indicating that OPN expression of damaged cells significantly increased. OPN expression on the cell surface after TPS treatment was higher than that of the normal group but less than that of the injury group, and OPN expression of the TPS2 group was the least. At the same time, the nuclei of the normal group were round, the nuclei of the injury group were deformed, and the nuclei of the protection group were mostly round and partially deformed. These results were consistent with the cell morphology observation in Figure 2.

The quantitative detection results of OPN expression (Figure 6(b)) were consistent with the qualitative observations. The OPN average fluorescence intensity of the normal group was approximately 29000, whereas it increased to 62000 in the injury group. The average fluorescence intensities of protection groups (47500, 38700, 33600, and 43000) were between those of the normal and injury groups.

### 3.7. PS Eversion

**Figure 7:**
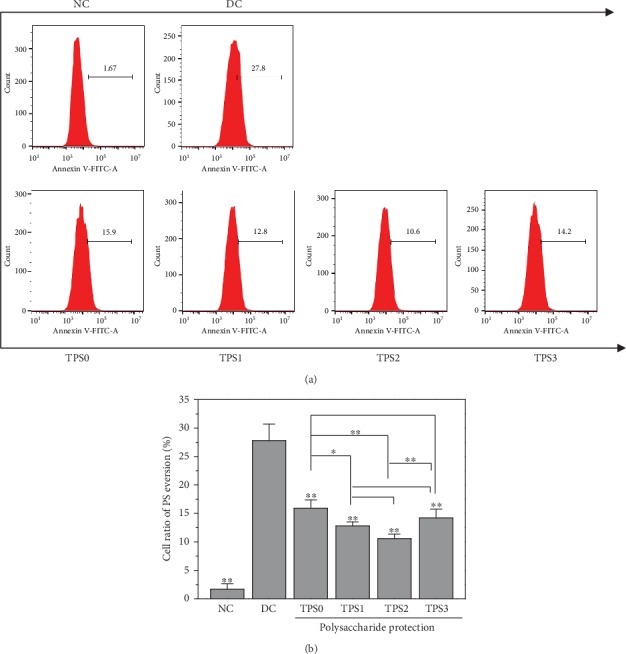
Effect of nano-COM crystals on the PS valgus of HK-2 cells before and after TPS protection: (a) quantitative histogram; (b) statistical histogram. Experimental conditions are the same as in Figure 1.

Figure 7 shows the effect of COM crystals on the cell surface PS eversion before and after TPS treatment. The amount of PS eversion in the normal group was low (1.67%), and it significantly increased (27.8%) in the injury group. PS eversions of protected cells were 15.9%, 12.8%, 10.6%, and 14.2%, and these values were between those of the normal and injury groups.

### 3.8. SEM Observation of Adherent Crystals on Cell Surface

**Figure 8:**
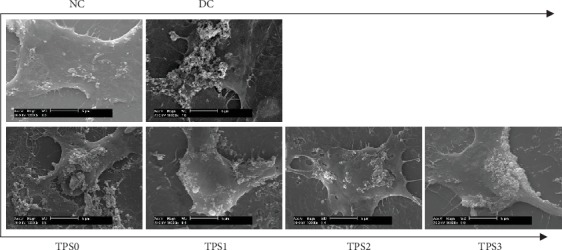
SEM images of nano-COM crystal adhesion on cells before and after TPS protection with different molecular weights. Experimental conditions are the same as those in Figure 1.

Figure 8 shows the SEM images of the adhesion of COM crystals on the cells before and after TPS treatment with different molecular weights. Few COM crystals adhered to the surface of normal cells. Damaged cells showed decreased morphology, and many crystals were found on the surface of these cells, thereby causing serious crystal aggregation. The cell morphology in TPS protection groups gradually recovered, and the adhesion amount and aggregation degree of crystals on the cell surface were lower than those in the damage group. For the TP2 protection group, its cell morphology recovered similar to normal cells with few adhered crystals, although the adhered crystals did not aggregate.

### 3.9. Percentage of Adhered Cells to Crystals

**Figure 9:**
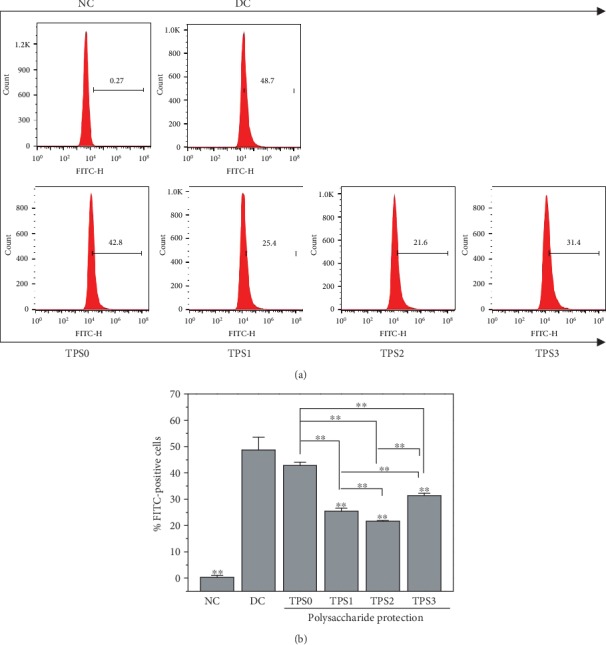
Percentage of cells adhering to nano-COM crystals before and after TPS protection: (a) quantitative histogram; (b) statistical histogram. Experimental conditions are the same as in Figure 1.

Figure 8 shows the percentage of adhered cells to crystals before and after TPS treatment. The percentage of cells in the injury group was 48.7%, whereas the percentage of cells in TPS0, TPS1, TPS2, and TPS3 groups were 42.8%, 25.4%, 21.6%, and 31.4%, respectively (Figure 9(b)). In other words, TPS treatment can inhibit the adhesion of COM crystals to cells.

## 4. Discussion

### 4.1. Reduction of Cytotoxicity of COM Crystals after Treatment of TPSs with Different Molecular Weights

Renal epithelial cell damage is a key factor that leads to the formation of kidney stones. Normal kidney tissues have an effective antioxidant defense system, such as superoxide dismutase (SOD) and other enzymes that can remove free radicals and their metabolites and protect the body from oxidative damage. In the pathological state, excessive free radicals produced in the body cause oxidative damage to kidney tissues, leading to the formation of diseases, such as kidney stones [22]. TPSs have good antioxidant capacity, and TPS treatment can improve the ability of renal epithelial cells to resist oxidative damage [23, 24].

The results showed that TPSs protected HK-2 cells from damage caused by nano-COM crystals by reducing ROS production. After the TPS treatment, mitochondrial membrane potential and lysosomal integrity improved, cell membrane damage was reduced, and cell morphology was repaired.

Many studies have demonstrated the protective effect of PSs on cells. For example, Jia et al. [14] observed that low-molecular weight (7 kDa) fucoidan could protect renal function and tubular cells from albumin overload caused by injury and inhibit the increase of ROS levels. Li et al. [15] showed that *Lycium barbarum* PS (LBP-4a) exhibits protective effects against renal damage induced by KBrO_3_. Its mechanism was closely correlated with the reduction in lipid peroxidation levels and the increase in the activities of antioxidant enzymes in kidney tissues, which alleviated DNA damage and increased mitochondrial membrane potentials in renal cells. Zhang et al. [16] showed that the viability and morphology of rat bone marrow endothelial progenitor cells decrease after thrombin damage, and *Astragalus* PS (APS) inhibited this damage.

The molecular weight of TPSs affected their protective effect on HK-2 cells. The cell membrane cannot be crossed when the molecular weights of the TPSs were large (e.g., 10.88 kDa of TPS0) [25, 26]. However, the hydrogen chain was weak, and the active helical structure was not formed because of the short sugar chain [27, 28] when the molecular weight of TPSs was small (such as 2.31 kDa of TPS3), thereby leading to a decrease in the protective effect. Thus, TPS2 with moderate molecular weight (4.82 kDa) was the most active. The molecular weight ranges of different PSs that show strong biological activity differ because of their monosaccharide composition, acidic group species, and content. For example, Yuan et al. [29] demonstrated that all purified PSs obtained from *Ligusticum chuanxiong* Hort (LCA, LCB, and LCC at approximately 2.83 × 10^4^, 1.23 × 10^4^, and 6.31 × 10^4^ Da, respectively) exhibit antioxidant properties and cytotoxicity. Among these, LCB had the highest antioxidant and cytotoxic activity. You et al. [30] investigated the protective effects of *Lentinus edodes* PSs with molecular weights of 25.5, 306.2, and 605.4 kDa on D-galactose-induced oxidative stress-induced myocardial cells in mice and showed that PSs with medium molecular weight have the strongest protective effect.

### 4.2. Inhibition of Adhesion of COM Crystals to HK-2 Cells after TPS Treatment

Crystallites are common compared with stones, thereby suggesting that crystal adhesion is the key to the formation of kidney stones [31, 32]. Crystallites are excreted in urine rather than adhering to renal epithelial cells and do not form kidney stones. Urine crystallites adhere to the surface of renal epithelial cells and grow or aggregate on the cell surface to form large-sized crystals, which lead to the formation of kidney stones [33].

The risk of stone formation increases, because cell damage promotes cell adhesion to CO crystals. For example, the adhesion of COM crystals to the surface of normal Madin-Darby canine kidney (MDCK) cells is 0.2 ± 0.03 *μ*g/cm^2^, and the surface of damaged MDCK increases to 3.90 ± 0.35 *μ*g/cm^2^, in which >90% of COM crystals adhere to damaged cells. After the repair of damaged cells, adhesion is reduced to 0.16 ± 0.02 *μ*g/cm^2^ [34].

After TPS treatment, the expression of negatively charged adhesion molecules, such as OPN (Figure 6) and PS (Figure 7), is inhibited due to the inhibition of cell damage [35, 36], thereby reducing the adhesion of nanocrystals (Figures 7 and 9). Veena et al. [37] found that fucoidan can protect the kidney of mice with high oxalic aciduria from damage; increase the activity of SOD, CAT, and GPX; prevent renal cell membrane damage; inhibit the adhesion of CO crystals; and reduce the deposition of CO crystals in mice.

The acid group-rich TPSs can adsorb on the surface of COM crystals, thereby increasing the absolute value of zeta potential on the crystal surface and reducing the adhesion of crystals to the surface of negatively charged cell surface. Verkoelen et al. [38] observed that natural glycosaminoglycans and semisynthetic PSs can inhibit the binding of COM crystals to the monolayers of MDCK cells. de Cógáin et al. [39] confirmed that the extract of *Costus arabicus* L. (*C. arabicus*), which contains a PS as its active ingredient, has no effect on the pretreatment of MDCK cells on crystal adhesion, and the pretreatment of COM crystals can significantly reduce crystal adhesion in a concentration-dependent manner. The adhesion amount was 0.95 ± 0.12 when the concentration of *C. arabicus* was 1 *μ*g/mL compared with the normal group, and the adhesion amount decreased to 0.37 ± 0.1 when the concentration increased to 1000 *μ*g/mL. This condition was similar to the inhibitory effect of heparin in the positive control group.

**Figure 10:**
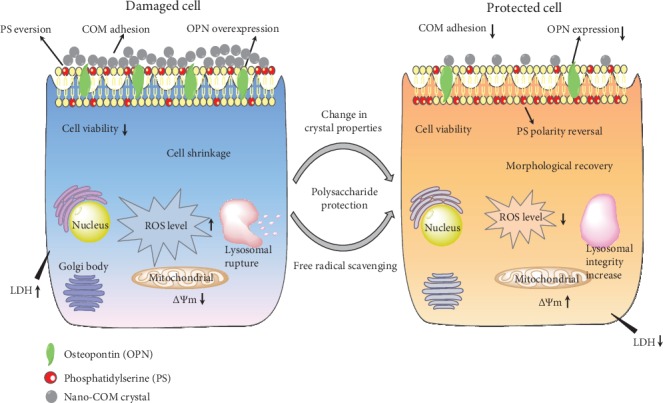
Inhibition of adhesion of HK-2 cells to nano-COM crystals by TPS protection.

Figure 10 shows the inhibition model of adhesion of HK-2 cells to nano-COM crystals after TPS treatment. TPS treatment can reduce the zeta potential of the crystal surface and the amount of exposed Ca^2+^, thereby inhibiting the adhesion of crystals to negatively charged cells on the surface. In addition, PSs can inhibit cell membrane damage by eliminating excess ROS, increasing mitochondrial membrane potential, improving lysosomal integrity, repairing cell morphology, reducing OPN expression, inhibiting PS eversion, and inhibiting crystal adhesion.

## 5. Conclusion

Treatment with TPSs with different molecular weights can inhibit the damage of nano-COM crystals to HK-2 cells, improve cell viability, reduce LDH release, restore cell morphology, reduce ROS level, improve mitochondrial membrane potential, improve lysosomal integrity, reduce OPN expression, inhibit PS eversion, and reduce crystal adhesion. The protective effect of TPSs is related to their molecular weight. TPS2 with moderate molecular weight has the best protective effect and is a potential drug for preventing stones.

## Data Availability

All the data supporting the results are shown in the paper and can be obtained from the corresponding authors.
